# Supplementary material for: Effects of an invasive predator cascade to plants via mutualism disruption
Source: Nat Commun. 2017 Mar 8;8:14557. doi: 10.1038/ncomms14557 (PMC5344968; doi:10.1038/ncomms14557)
Supplement: Supplementary Information — Supplementary Figures, Supplementary Tables and Supplementary References [file ncomms14557-s1.pdf]

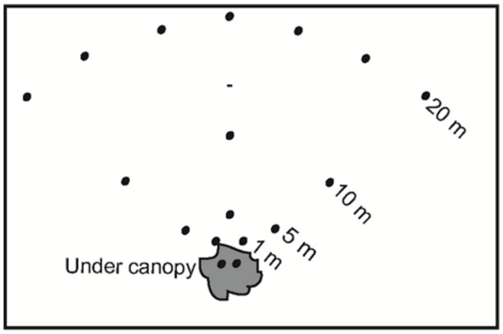


**Supplementary Figure 1| Seed trap array configuration**: The focal tree is in grey; other trees present at each site were mapped and included in the analysis, but are not shown here. Each black dot represents a seed trap.

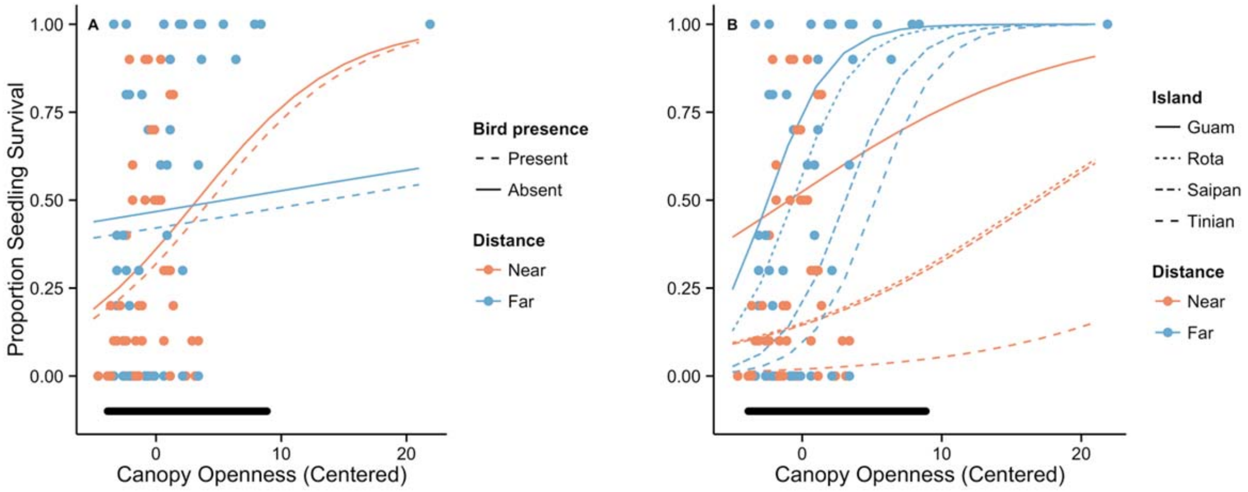


**Supplementary Figure 2| Distance-dependent mortality**: Seedling survival of a) *Psychotria* and b) *Premna* near (orange) and far (blue) from conspecific trees on islands with (dashed lines) and without (solid line) dispersers, as a function of canopy openness (centered so a value of zero corresponds to the mean openness of 9.12%). Model fits (lines) are plotted over observed data. Bars at the bottom of each panel indicate the 95% quantiles of canopy openness across all species and site.

**Supplementary Table 1: Seed Dispersers in the Marianas.** Distribution of avian seed dispersers in the Mariana Islands, and whether each species has been observed consuming *Psychotria* or *Premna* fruits in the wild during >100 hours of fruiting tree observations per species. Data on the Mariana Crow diet is supplemented by observations reported in Faegre[1]. P=present and frugivory observations. P = currently present, HP = historically present but now extinct in the wild, A = absent.

| Species | Guam | Rota | Tinian | Saipan | Psychotria | Premna |
|---|---|---|---|---|---|---|
| Micronesian Starling (*Aplonis opaca*) | **P** | **P** | **P** | **P** | Yes | Yes |
| Mariana Fruit Dove (*Ptilinopus roseicapilla)* | **HP** | **P** | **P** | **P** | Yes | Yes |
| White-throated Ground Dove *(Gallicolumba xanthonura)* | **HP** | **P** | **P** | **P** | No | Yes |
| Bridled White-eye (*Zosterops conspicillatus*) | **HP** | A | **P** | **P** | Yes | Yes |
| Rota Bridled White-eye (*Zosterops rotensis*) | A | **P** | A | A | No data | No data |
| Mariana Crow (*Corvus kubaryi*) | **HP** | **P** | A | A | Yes | Yes |
| Golden White-eye (*Cleptornis marchei*) | A | A | **HP** | **P** | Yes | Yes |

**Supplementary Table 2: Proportion Seeds Ingested model coefficients.** Results from species-specific generalized linear mixed-effects models comparing the effect of island on the proportion seeds ingested for each plant species. Upon confirmation that the best-fitting model included island (based on AICc comparison with a null model; Psychotria $\Delta AIC_c$ = 11.48; *Premna* $\Delta AIC_c$ = 11.40), we estimated 95% confidence intervals around the coefficients for each island using profile likelihood. Guam was set as the reference level for these treatment contrasts. Confidence intervals that do not include zero are in bold.

| Factor Level | Coefficient (lower, upper CI) |
|---|---|
| | *Psychotria* |
| **Intercept (Guam)** | **-4.13 (-5.59, -2.98)** |
| **Saipan** | **4.33 (2.71, 6.27)** |
| **Tinian** | **2.85 (1.23, 4.79)** |
| **Rota** | **3.32 (1.66, 5.38)** |
| | *Premna* |
| **Intercept (Guam)** | **-2.76 (-3.74, -1.80)** |
| **Saipan** | **3.84 (2.51, 5.33)** |
| **Tinian** | **2.24 (0.87, 3.63)** |
| **Rota** | **2.92 (1.55, 4.31)** |

**Supplementary Table 3: Germination Experiment model coefficients.** Results from species-specific generalized linear models comparing the effect of seed condition (ingested by birds, mechanically de-pulped, whole fruit) on germination. Upon confirmation that the best-fitting model included treatment (based on $AIC_c$ comparison with a null model; *Psychotria* $\Delta AIC_c$ = 31.05, *Premna* $\Delta AIC_c$ = 33.5), we estimated 95% confidence intervals around the coefficients for each treatment using profile likelihood, first using treatment contrasts with 'whole fruit' as the reference level, and then using treatment contrasts with 'pulp manually removed' as the reference level. Confidence intervals that do not include zero are in bold.

| Reference Level | Factor Level | Coefficient (lower, upper CI) |
|---|---|---|
| | *Psychotria* | |
| Whole fruit | **Intercept** | **-1.85 (-2.34, -1.42)** |
| | **Pulp removed** | **0.65 (0.05, 1.26)** |
| | **Ingested** | **2.05 (1.52, 2.62)** |
| Pulp Removed | **Intercept** | **-1.20 (-1.61, -0.83)** |
| | **Ingested** | **1.40 (0.92, 1.90)** |
| | *Premna* | |
| Whole fruit | **Intercept** | **-0.54 (-0.93, -0.17)** |
| | Pulp removed | 0.11 (-0.43, 0.66) |
| | **Ingested** | **1.39 (0.88, 1.91)** |
| Pulp Removed | **Intercept** | **-0.43 (-0.83, -0.04)** |
| | **Ingested** | **1.28 (0.75, 1.81)** |

**Supplementary Table 4: Seed Ingestion Relative to Distance model coefficients.** Results from generalized linear mixed-effects models examining how the proportion of seeds ingested varies with the distance to the nearest conspecific tree. Only data from Saipan, Tinian, and Rota were included, as few seeds were found >5 m from a conspecific tree on Guam. Upon confirmation that the best-fitting model included an island by distance interaction (based on AICc comparison with all sub-models; Psychotria $\Delta AIC_c$ =25.38; *Premna* $\Delta AIC_c$ =91.65), we estimated 95% confidence intervals around the coefficients for each island using profile likelihood. Rota was set as the reference level for these treatment contrasts. Confidence intervals that do not include zero are in bold.

| Factor Level | Coefficient (lower, upper CI) |
|---|---|
| | *Psychotria* |
| **Intercept (Rota)** | **-2.30 (-3.12, -1.26)** |
| Saipan | 1.20 (-0.12, 2.26) |
| Tinian | 0.45 (-0.87, 1.50) |
| **Rota×Distance** | **0.46 (0.35, 0.58)** |
| Saipan×Distance | 0.12 (-0.02, 0.26) |
| **Tinian×Distance** | **-0.15 (-0.29, -0.03)** |
| | *Premna* |
| Intercept (Rota) | 0.28 (-0.97, 1.52) |
| Saipan | 0.04 (-1.68, 1.89) |
| Tinian | -1.51 (-3.28, 0.24) |
| Rota×Distance | -0.03 (-0.08, 0.02) |
| **Saipan×Distance** | **0.27 (0.21, 0.32)** |
| **Tinian×Distance** | **0.28 (0.22, 0.34)** |

**Supplementary Table 5: Dispersal Kernel model selection.** Model selection for dispersal kernels fitted to seed trap data for two tree species on Guam and three nearby islands where native frugivorous birds are present. Kernels were either power-exponential (PE) or 2D*t*, and the scale parameter was either identical for all islands (no island effects), distinct for Guam but identical on the other islands, or distinct for each island. All models include random site-level variation in tree fecundity per basal area. For each species, models are ranked in order of decreasing strength of evidence. MCMC output is summarized as posterior mean deviance ($\overline{D(\theta)}$), effective parameter dimension ($p_V$), differences in deviance information criterion (ΔDIC), model weight ($w_{DIC}$), and the posterior mean squared correlation ($R^2$) between fitted and observed log(seeds + 1).

| **Kernel** | **Island effects** | $\overline{D(\theta)}$ | $p_V$ | **ΔDIC** | $w_{DIC}$ | $R^2$ |
|---|---|---|---|---|---|---|
| | | *Psychotria* | | | | |
| 2Dt | Guam vs. others | 1368.49 | 23.99 | 0.00 | 0.845 | 0.59 |
| 2Dt | all distinct | 1367.47 | 29.00 | 3.99 | 0.115 | 0.60 |
| PE | Guam vs. others | 1376.17 | 23.64 | 7.33 | 0.022 | 0.59 |
| PE | all distinct | 1374.04 | 26.16 | 7.72 | 0.018 | 0.59 |
| 2Dt | none | 1392.96 | 22.62 | 23.10 | 0.000 | 0.57 |
| PE | none | 1396.07 | 24.91 | 28.51 | 0.000 | 0.55 |
| | | *Premna* | | | | |
| PE | Guam vs. others | 1771.85 | 26.10 | 0.00 | 0.903 | 0.39 |
| PE | all distinct | 1771.17 | 31.46 | 4.68 | 0.087 | 0.39 |
| 2Dt | Guam vs. others | 1782.42 | 24.79 | 9.26 | 0.009 | 0.37 |
| 2Dt | all distinct | 1780.86 | 31.12 | 14.03 | 0.001 | 0.37 |
| 2Dt | none | 1795.89 | 22.69 | 20.63 | 0.000 | 0.40 |
| PE | none | 1802.48 | 24.86 | 29.39 | 0.000 | 0.38 |

**Supplementary Table 6: Dispersal Kernel parameter estimates.** Parameter estimates (posterior means and 95% credible intervals) for dispersal kernels fitted to seed trap data for two tree species on Guam and three nearby islands where native frugivorous birds are present. Kernels were either power-exponential (PE) or 2D*t*, and the scale parameter was either identical for all islands (no island effects), distinct for Guam but identical on the other islands, or distinct for each island. All models include site-level variation in tree fecundity per basal area as a lognormally distributed random effect with log-mean $\mu_\beta$ and SD $\sigma_\beta$. The island-specific kernel scale parameters for Guam, Rota, Saipan, and Tinian are $a_G$, $a_R$, $a_S$, and $a_T$ respectively; note that some of these may be identical depending on the model structure. The negative binomial likelihood includes variance parameters $k_1$ and $k_2$.

| Kernel | Island effects | $\mu_\beta$ | $\sigma_\beta$ | $a_G$ | $a_R$ | $a_S$ | $a_T$ | $k_1$ | $k_2$ |
|---|---|---|---|---|---|---|---|---|---|
| | | | | | *Psychotria* | | | | |
| 2Dt | Guam vs. others | 4.22 (3.38, 5.06) | 1.17 (0.57, 2.14) | 0.46 (0.19, 0.86) | 3.42 (2.49, 4.61) | 3.42 (2.49, 4.61) | 3.42 (2.49, 4.61) | 7.13 (2.02, 19.42) | 2.02 (1.48, 2.62) |
| 2Dt | all distinct | 4.3 (3.5, 5.05) | 0.98 (0.44, 1.83) | 0.46 (0.21, 0.84) | 7.81 (3.01, 21.4) | 3.91 (2.5, 5.78) | 2.49 (1.5, 3.82) | 9.45 (2.38, 27.4) | 1.93 (1.33, 2.55) |
| PE | Guam vs. others | 4.06 (3.27, 4.76) | 1.1 (0.55, 2) | 0.05 (0.03, 0.09) | 0.28 (0.19, 0.43) | 0.28 (0.19, 0.43) | 0.28 (0.19, 0.43) | 10.03 (2.18, 30.96) | 1.96 (1.36, 2.59) |
| PE | all distinct | 4.17 (3.42, 4.82) | 0.96 (0.46, 1.74) | 0.05 (0.03, 0.08) | 0.8 (0.24, 2.28) | 0.31 (0.19, 0.53) | 0.17 (0.1, 0.31) | 11.47 (2.45, 32.9) | 1.88 (1.26, 2.52) |
| 2Dt | none | 3.95 (2.93, 4.94) | 1.57 (0.86, 2.67) | 2.19 (1.5, 2.93) | 2.19 (1.5, 2.93) | 2.19 (1.5, 2.93) | 2.19 (1.5, 2.93) | 4.48 (1.22, 12.23) | 2.43 (1.84, 3.13) |
| PE | none | 3.94 (3.03, 4.76) | 1.34 (0.71, 2.34) | 0.21 (0.14, 0.38) | 0.21 (0.14, 0.38) | 0.21 (0.14, 0.38) | 0.21 (0.14, 0.38) | 14.31 (1.59, 75.07) | 2.19 (1, 3.04) |

| | | | | | | | | | |
|---|---|---|---|---|---|---|---|---|---|
| | | | | *Premna* | | | | | |
| PE | Guam vs. others | 3.74 (2.55, 4.95) | 1.93 (1.11, 3.23) | 0.06 (0.04, 0.09) | 0.41 (0.23, 0.74) | 0.41 (0.23, 0.74) | 0.41 (0.23, 0.74) | 3.09 (1.04, 11.07) | 3 (2.47, 3.64) |
| PE | all distinct | 3.94 (2.75, 5.09) | 1.84 (1.02, 3.13) | 0.06 (0.04, 0.09) | 1.13 (0.07, 9.2) | 0.71 (0.25, 2.08) | 0.39 (0.19, 0.88) | 3.02 (1.03, 10.47) | 3 (2.45, 3.67) |
| 2Dt | Guam vs. others | 3.82 (2.65, 5.07) | 1.91 (1.09, 3.26) | 0.86 (0.21, 1.78) | 4.78 (3.08, 7.41) | 4.78 (3.08, 7.41) | 4.78 (3.08, 7.41) | 3.94 (1.01, 14.88) | 3.18 (2.6, 3.86) |
| 2Dt | all distinct | 3.98 (2.74, 5.43) | 1.89 (1.04, 3.31) | 0.82 (0.21, 1.71) | 8.26 (0.63, 76.97) | 7.55 (3.74, 15.04) | 4.3 (2.18, 7.63) | 3.96 (1.03, 14.43) | 3.15 (2.57, 3.83) |
| 2Dt | none | 3.67 (2.34, 4.93) | 2.14 (1.27, 3.54) | 2.72 (1.79, 3.76) | 2.72 (1.79, 3.76) | 2.72 (1.79, 3.76) | 2.72 (1.79, 3.76) | 2.64 (0.94, 8.35) | 3.4 (2.77, 4.16) |
| PE | none | 3.72 (2.46, 5.01) | 2.13 (1.26, 3.63) | 0.19 (0.13, 0.28) | 0.19 (0.13, 0.28) | 0.19 (0.13, 0.28) | 0.19 (0.13, 0.28) | 2.82 (0.93, 9.4) | 3.48 (2.84, 4.29) |

**Supplementary Table 7: Dispersal kernel summary statistics.** Summary statistics for the marginal distribution of seed dispersal distance (m) away from the parent tree. Estimates are based on dispersal kernels fitted to seed trap data for two species on Guam and three nearby islands where native frugivorous birds remain. The best-supported kernel model for each species was used to calculate the mean and mode of dispersal distance and the probability that a seed lands beneath the canopy of the parent (a distance of 2 m or 4 m for *Psychotria* or *Premna* respectively, based on the typical crown radius of trees measured at our study sites). Posterior means are shown, with 95% credible intervals in parentheses.

| **Kernel** | **Island** | **Mean** | **Mode** | **P($r < r_c$)** |
|---|---|---|---|---|
| | | *Psychotria* | | |
| 2Dt | Guam | 0.73 (0.29, 1.35) | 0.63 (0.38, 0.92) | 0.94 (0.84, 0.99) |
| 2Dt | islands with birds | 5.37 (3.91, 7.24) | 2.03 (1.69, 2.42) | 0.26 (0.16, 0.39) |
| | | *Premna* | | |
| PE | Guam | 1.18 (0.78, 1.74) | 0.24 (0.16, 0.35) | 0.96 (0.91, 0.99) |
| PE | islands with birds | 8.19 (4.63, 14.87) | 1.64 (0.93, 2.97) | 0.40 (0.20, 0.60) |

**Supplementary Table 8: Model comparison for seedling distance-dependent mortality study.** Model comparison for generalized linear mixed-effects models examining the effect of distance (near/far), bird presence (yes/no) or island, canopy openness, and the interaction between distance and canopy openness and distance and bird presence/island on seedling survival of *Psychotria* and *Premna*.

| Model | $\Delta AIC_c$ | # parameters | $AIC_c$ weights |
|---|---|---|---|
| | *Psychotria* | | |
| Distance×Canopy + Bird | 0 | **6** | **0.73** |
| Distance×Canopy + Distance×Bird | 2.34 | **7** | **0.23** |
| Distance×Canopy + Distance×Island | 7.71 | **9** | **0.02** |
| Distance + Canopy | 8.05 | **4** | **0.01** |
| Canopy | 9.95 | **3** | **0.01** |
| Distance + Canopy + Bird | 10.19 | **5** | **0.00** |
| Canopy + Bird | 12.08 | **4** | **0.00** |
| Distance | 18.82 | **3** | **0.00** |
| Distance + Bird | 20.19 | **4** | **0.00** |
| Bird | 22.47 | **3** | **0.00** |
| | *Premna* | | |
| Distance×Canopy + Distance×Island | 0 | **11** | **0.61** |
| Distance×Canopy + Island | 1.17 | **8** | **0.34** |
| Distance×Canopy + Distance×Bird | 5.44 | **7** | **0.04** |
| Distance + Canopy + Island | 8.81 | **7** | **0.01** |
| Distance + Canopy | 19.67 | **4** | **0** |
| Canopy + Island | 40.04 | **6** | **0** |
| Canopy | 50.41 | 3 | 0 |
| Distance + Island | 134.24 | **6** | **0** |
| Distance | 148.95 | **3** | **0** |
| Island | 201.23 | **5** | **0** |

**Supplementary Table 9: Seedling distance-dependent mortality model coefficients.** Estimated coefficients and confidence intervals for the best-fitting generalized linear mixed-effects model examining the effect of distance (near/far), bird presence (yes/no) or island, and canopy openness on seedling survival of *Psychotria* and *Premna*. We used profile likelihoods to estimate 95% confidence intervals around the coefficients, based on treatment contrasts with Guam (no birds) and distance=near as the reference levels. The two species (*Psychotria* and *Premna*) were analyzed separately. Confidence intervals that do not include zero are in bold.

| **Factor Level** | **Coefficient (lower, upper CI)** |
|---|---|
| *Psychotria* | |
| Intercept (Distance = Near, Bird = Yes) | **-0.76 (-1.28, -0.26)** |
| Distance = Far | **0.45 (0.09, 0.80)** |
| Canopy openness | **0.18 (0.10, 0.26)** |
| Bird = No | 0.19 (-0.63, 1.00) |
| Distance = Far × Canopy | **-0.15 (-0.25, -0.06)** |
| *Premna* | |
| Intercept (Distance = Near, Island = Guam) | 0.09 (-0.70, 0.89) |
| Island = Rota | **-1.81 (-3.17, -0.48)** |
| Island = Saipan | **-1.87 (-3.21, -0.55)** |
| Island = Tinian | **-4.00 (-6.32, -2.15)** |
| Distance = Far | **1.01 (0.52, 1.5)** |
| Canopy | 0.10 (-0.06, 0.25) |
| Distance = Far × Canopy | **0.36 (0.17, 0.55)** |
| Island = Rota × Distance = Far | **0.86 (0.002, 1.73)** |
| Island = Saipan × Distance = Far | -0.62 (01.49, 0.26) |
| Island = Tinian × Distance = Far | 0.61 (-1.02, 2.63) |

**Supplementary Table 10: Model comparison for study on distance-dependent mortality during germination and early seedling survival stages.** Model comparison for generalized linear models examining the effect of distance (near/far), canopy openness, and the study site on germination and early seedling survival of *Premna*.

| **Model** | **$\Delta AIC_c$** | **# parameters** | **$AIC_c$ weights** |
|---|---|---|---|
| | Germination | | |
| Distance | 0 | **2** | **0.48** |
| Distance + Site | 1.98 | **4** | **0.18** |
| Distance + Site + Canopy | 3.25 | **5** | **0.09** |
| Canopy | 3.31 | **2** | **0.09** |
| Site + Canopy | 4.52 | **4** | **0.05** |
| Null | 4.52 | **1** | **0.05** |
| Distance×Canopy + Site | 5.85 | **6** | **0.03** |
| Site | 5.97 | **3** | **0.02** |
| | Seedling Survival | | |
| Distance + Site + Canopy | 0 | **5** | **0.36** |
| Distance + Site | 0.53 | **3** | **0.28** |
| Distance | 1.10 | **2** | **0.21** |
| Distance×Canopy + Site | 2.03 | **6** | **0.13** |
| Canopy + Site | 5.85 | **4** | **0.02** |
| Canopy | 8.45 | **2** | **0.01** |
| Site | 8.51 | 3 | 0.01 |
| Null | 13.69 | **1** | **0.00** |

**Supplementary Table 11: Fitted parameters for distance-dependent mortality model focused on germination and early seedling survival .** Estimated coefficients and confidence intervals for the best-fitting generalized linear model examining the effect of distance (near/far), canopy openness, and site on germination and early seedling survival of *Premna*. We used profile likelihoods to estimate 95% confidence intervals around the coefficients, based on treatment contrasts with site=FORBI and distance=near as the reference levels. The two life stages (germination and early seedling survival) were analyzed separately. Confidence intervals that do not include zero are in bold.

| **Factor Level** | **Coefficient (lower, upper CI)** |
| --- | --- |
| Germination | |
| Intercept (Distance = Near, Site = FORBI) | **-2.75 (-3.04, -2.48)** |
| Distance = Far | **0.47 (0.12, 0.84)** |
| Seedling Survival | |
| Intercept (Distance = Near, Site = FORBI) | **-2.79 (-4.33, -1.64)** |
| Distance = Far | **1.72 (0.53,3.22)** |
| Canopy | 0.21 (-0.02, 0.46) |
| Site = LADT | -0.69 (-2.29, 0.66) |
| Site = MTR1 | 0.67 (-0.32, 1.78) |

**Supplementary Table 12: Integrative Metric of Bird Loss.** Predictions were generated by combining experimentally parameterized models of seed dispersal, seed ingestion by birds, seed germination, and seedling survival.

| | **Relative survival without birds** | |
|---|---|---|
| **Island** | ***Psychotria*** | ***Premna*** |
| Guam | 0.28 (0.17, 0.41) | 0.13 (0.05, 0.28) |
| Rota | 0.38 (0.24, 0.54) | 0.10 (0.03, 0.21) |
| Saipan | 0.28 (0.18, 0.41) | 0.13 (0.04, 0.29) |
| Tinian | 0.39 (0.25, 0.54) | 0.08 (0.02, 0.23) |

## Supplementary References

1.Faegre, S. K. Age-related differences in diet and foraging behavior of the critically endangered Mariana Crow (Corvus kubaryi). (2014). Masters Thesis, University of Washington. Seattle. 35 p.
